# Increasing the Impact of JMIR Journals in the Attention Economy

**DOI:** 10.2196/16172

**Published:** 2019-10-31

**Authors:** Ricky Leung

**Affiliations:** 1 University at Albany School of Public Health Rensselaer, NY United States

**Keywords:** JMIR, medical informatics, digital health, publishing, knowledge translation, peer-to-peer community, impact

## Abstract

The Journal of Medical Internet Research (JMIR) has attained remarkable achievements in the past twenty years. By depth, JMIR has published the most impactful research in medical informatics and is top ranked in the field. By width, JMIR has spun off to about thirty sister journals to cover topics such as serious games, mobile health, public health, surveillance, and other medical areas. With ever-increasing data and research findings, academic publishers need to be competitive to win readers’ attention. While JMIR is well-positioned in the field, the journal will need more creative strategies to increase its attention base and maintain its leading position. Viable strategies include the creation of online collaborative spaces, the engagement of more diverse audience from less traditional channels, and partnerships with other publishers and academic institutes. Doing so could also enable JMIR researchers to turn research insights into practical strategies to improve personal health and medical services.

The Journal of Medical Internet Research (JMIR) has engaged many health researchers since the journal published its inaugural issue in August 1999 [1]. JMIR is now ranked number one in the field of medical informatics, and the JMIR publishing office has issued close to 30 additional sister journals, including JMIR mHealth and uHealth, the Journal of Serious Games, and others. These are remarkable achievements. In this short article, I provide a quick review to highlight the important role of JMIR in promoting innovative research in the field of medical informatics. Building on its achievements, JMIR is well-positioned not only to maintain its leading position in medical informatics but also to contribute excellent research to the academic community at large.

Medical informatics researchers have long recognized that the internet is a big treasure of useful data to improve medical practice and health behaviors. For example, JMIR research published in earlier years studied how practitioners could utilize available statistical data on the internet to measure health quality [2-4]. As online tools became accessible, researchers analyzed whether the internet could serve as an effective platform for primary data collection through surveys [5,6], experiments [7], and even interviews [8]. Some researchers evaluated the efficacy of online medical treatment and found positive results in terms of reducing depression and other medical symptoms [9,10]. This research has paved the way for the emerging subfield of real-world data/evidence [11]. Pharmaceutical companies and public health agencies, such as the US Food and Drug Administration, have expended considerable efforts to promote real-world evidence to supplement and even replace expensive clinical trials.

JMIR research has applied some of the most sophisticated methods in the field. For instance, some researchers examined unstructured data with advanced text-mining techniques [12]. This line of research can detect the sentiments of participants in social media platforms [13] and can determine other deep meanings embedded in qualitative data. In addition, researchers are now able to build robots [14] and apply artificial intelligence [15] to conduct research projects that were not feasible in the past, such as using machine learning algorithms to capture real-time data from social media channels [16]. To reduce the impact of fake news on health outcomes, other JMIR researchers evaluated the veracity of news reports from multiple channels [17].

The rapid development of connected devices, such as wearable technology, smart appliances, and body sensors, has presented new opportunities and challenges for medical informatics research. JMIR has already published some exciting research about the Internet of Things which has multilevel implications for patients and health providers. For instance, many recent studies published in JMIR have shown a strong patient-centered orientation [18]. These studies focused on how the internet has enabled patients to incorporate first-hand experience into research to increase its practical value, whether from actual health care experience, usage of Internet-based devices, or information from peer groups. Funding agencies such as the Patient-Centered Outcomes Research Institute and the National Institutes of Health have paid attention to this research published in JMIR [19].

Beyond personal health, new information technology has improved the quality of health care delivery. For example, interorganizational networks have allowed hospitals to access patients’ records from different healthcare settings, facilitating the transition from electronic medical records to electronic health records [20]. Many studies published in JMIR were products of collaborative networks in health care. More recently, research published in JMIR has built ambitious frameworks useful for studying issues at the community and societal levels. Researchers have used these new frameworks to study such important global issues as aging [21], climate change [22], poverty [23] and sustainability [24].

The availability of big data in the medical sector has led to the challenge of limited attention for researchers [25]. While research institutes and established journals have employed aggressive marketing campaigns to attract attention from existing and potential consumers [26], medical researchers now receive enormous amounts of information from social media, personal devices, and other online platforms. In this context, JMIR needs to come up with creative strategies to increase its intellectual breadth and depth to maintain its leading position in the field. One viable strategy is to provide more collaborative space for JMIR subscribers and potential authors. That is, instead of serving only as a publisher of completed research, JMIR could also become a platform to promote collaboration for early-stage research. In fact, JMIR has recently started a Digital Health Community [27] within the JMIR Publications Knowledge Base and Help Center [28] to support the germination of new ideas and allow authors to provide feedback to the journal. The question is how to engage digital health researchers in platforms like that. To encourage increased participation in this community, JMIR may offer Karma points (already implemented to reward reviewers) that can serve as credits to reduce publication fees. Besides that, the JMIR editorial team can reach out and engage researchers from related fields to increase the journal’s impact. Increased attendance of academic conferences and professional meetings may be very useful for increasing the exposure of JMIR in various academic communities. Finally, JMIR may consider forming new partnerships with other publishers, professional organizations, academic institutes in different countries, and even funding agencies. Used properly, these strategies can increase the impact of JMIR in the next 20 years - beyond the impact factor.

## Abbreviations

JMIR: Journal of Medical Internet Research
